# High-intensity interval training attenuates urothelial nerve growth factor and angiotensin axis in hypertensive urinary bladder

**DOI:** 10.1038/s41440-026-02680-y

**Published:** 2026-05-20

**Authors:** Victor Rogério Garcia Batista, Maria Eduarda Almeida Tavares, Ivo Vieira de Sousa Neto, Rafael Jesus Gonçalves Rubira, Tatiana Aparecida de Oliveira, Allice Santos Cruz Veras, Antonio Hernandes Chaves-Neto, Francilene Lima Agostinho de Souza, Francis Lopes Pacagnelli, Vítor Samuel Leite Fernandes, Giovana Rampazzo Teixeira

**Affiliations:** 1 Multicenter Graduate Program in Physiological Sciences, SBFis, São Paulo State University (UNESP), Araçatuba, Brazil; 2 School of Physical Education and Sport of Ribeirão Preto, University of São Paulo (USP), Ribeirão Preto, Brazil; 3 Department of Physics, Institute of Geosciences and Exact Sciences (IGCE), (UNESP), Rio Claro, Brazil; 4 Institute of Chemistry, University of Campinas (UNICAMP), Campinas, Brazil; 5 Department of Basic Sciences, School of Dentistry, São Paulo State University (UNESP), Araçatuba, Brazil; 6 Postgraduate Animal Science Program, University of Western São Paulo (UNOESTE), Presidente, Brazil; 7 Departamento de Fisiología, Facultad de Farmacia, Universidad Complutense de Madrid, Madrid, Spain; 8 Department of Physical Education, São Paulo State University (UNESP), School of Technology and Sciences, Presidente Prudente, Brazil

**Keywords:** Spontaneously hypertensive rats, High-intensity interval training, Incremental hypertension, Spectroscopy Raman, Hypoxia

## Abstract

Arterial hypertension leads to urological complications by impairing urinary bladder function. Physical exercise, particularly high-intensity interval training (HIIT), is a non-pharmacological strategy for blood pressure control. HIIT improves oxygen consumption, body composition, and cardiovascular health, but its effects on the urinary bladder remain unclear. This study evaluated the effects of HIIT on structural and molecular markers of urinary bladder remodeling in spontaneously hypertensive rats (SHR), with emphasis on fibrosis, neurotrophic and angiogenic signaling, hypoxia-related pathways, and the balance between the ACE/Ang II/AT1R and ACE2/Ang-(1-7)/MasR axes. We also assessed its effects on blood pressure, metabolism, and redox biomarkers in blood circulation. HIIT significantly reduced serum glucose and lipid levels while improving redox balance with increased antioxidants. In the bladder, HIIT downregulated VEGF and NGF, reduced collagen deposition, and decreased TGF-β and SMAD2 expression. Additionally, HIIT modulated the angiotensin receptor axis (AT1/MAS ratio) and upregulated VEGF, promoting angiogenesis. Publicly available microarray data from ischemic and aging models support our findings, highlighting the role of TGF-β pathways in bladder fibrosis. This study reveals key intracellular mechanisms linking HIIT to redox balance, fibrosis, hypoxia, vascular remodeling, and angiotensin receptor modulation. Together, our findings suggest HIIT as a potential therapeutic approach for vascular and structural remodeling in the hypertensive urinary bladder.

**Figure:**
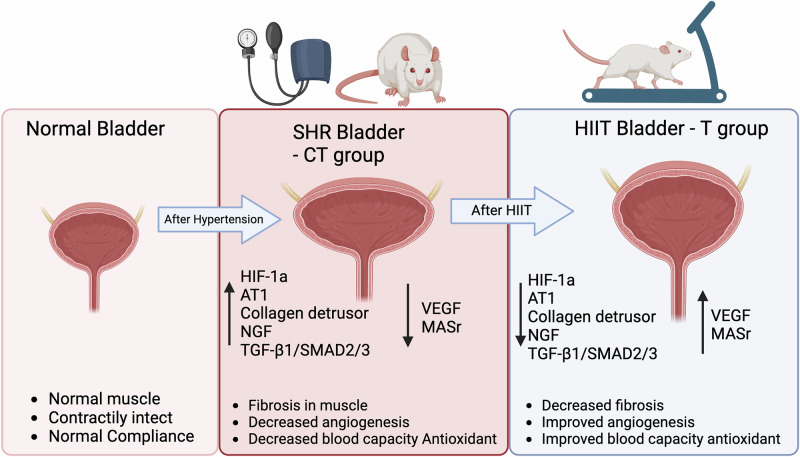
Impact of hypertension and HIIT on bladder physiology in SHR rats. The illustration depicts changes in bladder structure and protein modulation in spontaneously hypertensive rats (SHR) compared to normal controls, and the effects of an 8-week high-intensity interval training (HIIT) regimen. In normal bladder tissue, muscle structure and contractility are intact, with normal compliance. Following the onset of hypertension (SHR bladder), markers such as HIF-1α, AT1, detrusor collagen, NGF, and TGF-β1/SMAD2/3 are upregulated, leading to increased fibrosis, reduced contractility, and decreased bladder capacity, while VEGF, MAS receptor (MASr), and antioxidants are decreased. After HIIT (SHR + HIIT bladder), the bladder shows reduced fibrosis, improved contractility and increased capacity, accompanied by decreased expression of HIF-1α, AT1, detrusor collagen, NGF and TGF-β1/SMAD2/3, while VEGF, MASr and antioxidants are upregulated

Impact of hypertension and HIIT on bladder physiology in SHR rats. The illustration depicts changes in bladder structure and protein modulation in spontaneously hypertensive rats (SHR) compared to normal controls, and the effects of an 8-week high-intensity interval training (HIIT) regimen. In normal bladder tissue, muscle structure and contractility are intact, with normal compliance. Following the onset of hypertension (SHR bladder), markers such as HIF-1α, AT1, detrusor collagen, NGF, and TGF-β1/SMAD2/3 are upregulated, leading to increased fibrosis, reduced contractility, and decreased bladder capacity, while VEGF, MAS receptor (MASr), and antioxidants are decreased. After HIIT (SHR + HIIT bladder), the bladder shows reduced fibrosis, improved contractility and increased capacity, accompanied by decreased expression of HIF-1α, AT1, detrusor collagen, NGF and TGF-β1/SMAD2/3, while VEGF, MASr and antioxidants are upregulated

## Abstract (web summary)

Impact of hypertension and HIIT on bladder physiology in SHR rats. The illustration depicts changes in bladder structure and protein modulation in spontaneously hypertensive rats (SHR) compared to normal controls, and the effects of an 8-week high-intensity interval training (HIIT) regimen. In normal bladder tissue, muscle structure and contractility are intact, with normal compliance. Following the onset of hypertension (SHR bladder), markers such as HIF-1α, AT1, detrusor collagen, NGF, and TGF-β1/SMAD2/3 are upregulated, leading to increased fibrosis, reduced contractility, and decreased bladder capacity, while VEGF, MAS receptor (MASr), and antioxidants are decreased. After HIIT (SHR + HIIT bladder), the bladder shows reduced fibrosis, improved contractility and increased capacity, accompanied by decreased expression of HIF-1α, AT1, detrusor collagen, NGF and TGF-β1/SMAD2/3, while VEGF, MASr and antioxidants are upregulated.

## Introduction

Cardiovascular diseases are a major health concern globally, with arterial hypertension (AH) representing a significant portion of these diseases. The World Health Organization estimates that 1.28 billion adults aged 30 to 79 worldwide have hypertension [1]. AH is multifactorial, influenced by factors such as a sedentary lifestyle, a diet high in sodium, and obesity [2]. As age increases, the prevalence of AH rises, becoming a particular concern among the elderly. Furthermore, dysfunction of the lower urinary tract, including the urinary bladder, is a complication associated with AH, leading to harmful symptoms such as urgency, urinary frequency, and incomplete bladder emptying [3].

Arterial hypertension contributes to bladder dysfunction and reduced blood flow in the lower urinary tract. Studies indicate that detrusor muscle hyperactivity is associated with afferent hypertrophy and hypersensitivity, as well as a reduction in the number of cells in the greater pelvic ganglion [4]. It is well established that excessive production of Nerve Growth Factor (NGF) occurs in all smooth muscle of SHR animals, including the detrusor smooth muscle [5]. Increased NGF levels are associated with augmented neural excitation and a pronounced fibrotic profile, leading to hyperinnervation and bladder contractile dysfunction, which in turn exacerbates urinary frequency and impairs its functional capacity [6]. Chen et al. [7] reported that increased proliferation of smooth muscle cells and extracellular matrix (ECM) deposition contribute to bladder hypertrophy in a neurogenic model.

Neural and inflammatory alterations contribute to arterial hypertension, which is further sustained by Angiotensin II (AngII). AngII promotes oxidative stress and inflammation in the urinary bladder, triggering smooth muscle cell damage, fibrosis, and detrusor hypertrophy. The increase in AngII in spontaneously hypertensive rats (SHR) animals’ results in a decrease in total antioxidant capacity (TAC) and an increase in plasma hydrogen peroxide [8]. This occurs due to the activation of the AT-1 receptor, and an increase in NAD(P)H oxidase, promoting reactive oxygen species (ROS) production and systemic oxidative stress [9]. Furthermore, hypoxia upregulates Hypoxia-inducible factor 1-alpha (HIF-1α), triggering cell migration and proliferation [10]. Therefore, strategies aimed at reducing blood pressure, mitigating oxidative stress, and modulating inflammation are essential to prevent and control the detrimental effects of arterial hypertension on the urinary bladder microenvironment.

Among non-pharmacological strategies for the prevention and treatment of cardiovascular diseases [11], high-intensity interval training (HIIT) has gained significant attention due to its efficacy in improving cardiovascular function. The European and American Societies of Hypertension recommend various training modalities, among which HIIT has been proposed as an effective approach to reducing blood pressure and enhancing cardiovascular health [2]. HIIT consists of short bursts of intense exercise alternated with periods of active recovery, which has been shown to be superior to moderate-intensity continuous training (MICT) in promoting cardiovascular adaptations. Studies indicate that HIIT can lead to reductions of approximately 5 mmHg in systolic blood pressure and 3 mmHg in diastolic blood pressure in individuals with hypertension, with some meta-analyses suggesting even greater benefits in populations at higher cardiovascular risk [12]. The mechanisms underlying these effects include improved endothelial function, increased nitric oxide bioavailability, reduced arterial stiffness, and enhanced autonomic regulation of blood pressure [13]. Additionally, HIIT has been shown to reduce peripheral vascular resistance and improve blood flow, which may be relevant to urinary bladder function [14, 15]. These adaptations are particularly important for individuals with cardiovascular comorbidities, as they contribute to overall vascular health and organ perfusion. Furthermore, HIIT has been associated with increased insulin sensitivity, reduced inflammation, and favorable lipid profile changes, all of which contribute to cardiovascular risk reduction [16].

However, the molecular basis through which HIIT modulates hypertensive bladder remodeling remains insufficiently defined. Therefore, the present study investigated the effects of HIIT on key structural and signaling pathways involved in urinary bladder injury in SHR, including extracellular matrix deposition, TGF-β/SMAD-related fibrotic remodeling, NGF-associated neural signaling, local angiotensin receptor imbalance, and markers related to angiogenesis and hypoxia. In addition, we assessed circulating metabolic and redox parameters to determine whether local bladder changes occurred in parallel with broader systemic adaptations. Our results show that HIIT attenuated fibrotic remodeling, improved oxidative status, reduced hypoxia-related signaling, modulated neurotrophic and angiotensin-related pathways, and favored a more protective molecular profile in the urinary bladder. To strengthen the mechanistic interpretation of these findings, we also integrated publicly available transcriptomic datasets from chronic ischemia and aging models, which revealed convergent pathways related to extracellular matrix remodeling, fibrosis, hypoxia, and local renin–angiotensin system dysregulation.

## Materials and methods

### Animals and ethics statement

Twenty male spontaneously hypertensive rats (SHR) approximately 360 days old were obtained from the Central Vivarium of the State University of Campinas. The rats were housed in groups of 3–4 per plastic cage under controlled conditions (temperature: 21–23°C, humidity: 50–60%) with a 12-h light/dark cycle. The rats were randomly assigned to two experimental groups: spontaneously hypertensive rats (CT, *n* = 10) and spontaneously hypertensive rats subjected to high-intensity interval training (T, *n* = 10). Ethical approval for this study was obtained from the Ethics Committee for the Use of Animals (CEUA) of the São Paulo State University, Botucatu, under protocol number 1167/2016. All experimental procedures involving animals strictly followed the ethical guidelines of the Brazilian College of Animal Experimentation and complied with the *Guide for the Care and Use of Laboratory Animals* from the National Research Council.

### Systolic blood pressure (SBP)

Systolic blood pressure was assessed at baseline and after completion of the training protocol using a non-invasive tail-cuff plethysmography system (Narco Bio-System®, model 709-0610, International Biomedical, Inc., USA). To determine the impact of the intervention, changes in systolic blood pressure were expressed as percentage variation (Δ%), calculated by comparing post-training values to baseline using the following equation: [(post-training value − baseline value)/baseline value]×100. All measurements were performed according to standardized procedures previously established by our group [17].

### Maximus capacity test

**Fig. 1:**
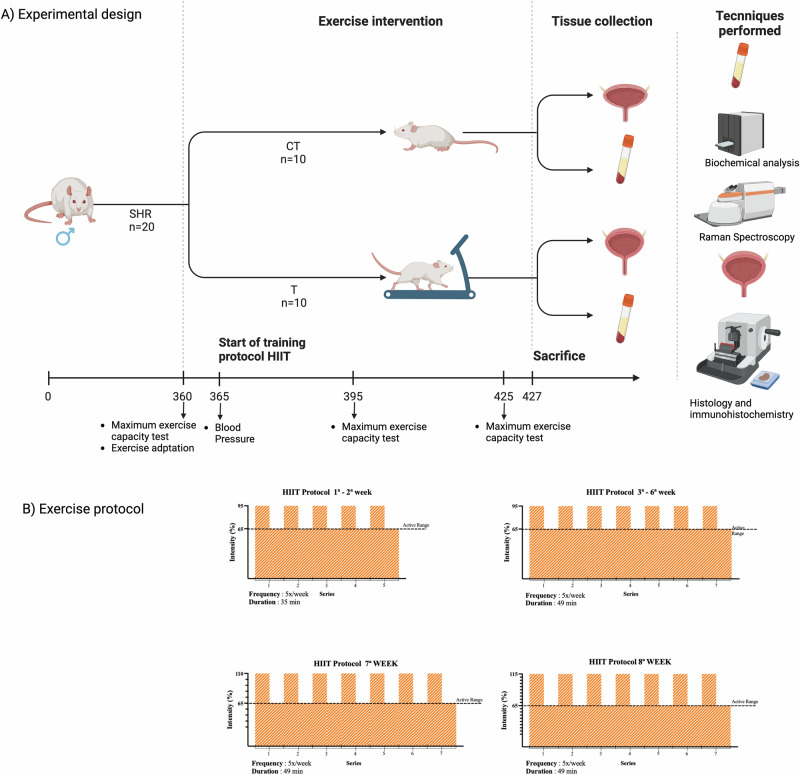
**A** Schematic representation of the experimental design and HIIT protocol in hypertensive rats (SHR). At 360 days of age, all animals underwent initial evaluations, including a maximal exercise capacity test on a treadmill and systolic blood pressure measurement. A 5-day adaptation period followed, during which the animals were familiarized with the treadmill and training protocol. At 365 days of age, animals were divided into two groups: CT (hypertensive Control) and T group (hypertensive trained), with the latter undergoing a high-intensity interval training (HIIT) protocol. At 395 days of age (start of the 4th training week), a second maximal exercise capacity test was conducted to adjust training intensity. At 425 days of age (end of the 8-week protocol), the final evaluations, including exercise performance tests, were performed. Animals were euthanized at 427 days of age, and tissue samples were collected for biochemical analysis, Raman spectroscopy, histology, and immunohistochemistry. **B** Graphical representation of the 8-week HIIT training protocol. During the 1st and 2nd weeks, the training consisted of 5 sets of 4 min at 95% of maximum speed, alternating with 3 min at 65% of maximum speed. From the 3rd to the 6th week, the intensity remained at 95%, but the number of sets increased to 7, maintaining the same alternation of 4 min at high intensity followed by 3 min at 65%. In the 7th week, the intensity was raised to 110%, with 7 sets of 4 min at this intensity, alternating with 3 min at 65%. In the 8th and final week, the intensity increased to 115%, maintaining the structure of 7 sets of 4 min at high intensity, alternating with 3 min at 65%

The animals underwent a five-day treadmill adaptation period using a TK 1 treadmill (Inbramed, São Paulo, Brazil), with each session lasting 10 min. Subsequently, a maximal aerobic capacity test was conducted on the treadmill without inclination. The test began at an initial speed of 6 m/min, with increments of 3 m/min every 3 min until the maximum speed for each animal was achieved. At each increment, the animals were monitored for total exhaustion. Maximum exhaustion was defined as occurring when a rat contacted the edge of the treadmill more than three times within one minute or exhibited changes in running biomechanics [17]. Functional capacity was assessed based on the total distance covered, calculated as the product of the speed and the maximum test duration for each animal. Evaluations were conducted at three points: before the start of physical training, after the fourth week of high-intensity interval training (HIIT) and following the final HIIT session (Fig. 1).

### High-intensity interval training protocol

The HIIT protocol was conducted over eight weeks, with daily sessions of approximately 50 min, five days per week. During the 5-day adaptation phase to the experimental HIIT protocol that has an estimated time of 50 min/day at 6 m/min, 5 days a week, for 8 weeks and a maximum capacity test to determine the speed of exhaustion, the protocol was performed as previously described [18], and training began with 5 sets of 4-min intervals at 95% of maximum speed, alternating with 3 min at 65% of maximum speed, during the first two weeks. In weeks three and four, the number of sets increased to 6 m/min per session while maintaining the same intensities. Before the fifth week, a new maximal capacity test was performed to readjust training speeds. In weeks five and six, the protocol was adapted to include 7 sets, maintaining intensities of 95% for 4 min and 65% for 3 min. In addition, intervals at 115% of maximum speed were introduced, alternating with 3-min periods at 65%, completing seven sets. This progressive protocol was designed to optimize cardiovascular adaptation and physical performance throughout the training period (Fig. 1).

### Biochemical analyzes of plasmatic redox state

At 427 days of age, 48 h after the final HIIT session, the animals were anesthetized with ketamine (50 mg/kg, intraperitoneally) and xylazine (10 mg/kg, intraperitoneally), then euthanized via decapitation. Trunk blood was promptly collected into heparinized tubes. Following centrifugation, serum was isolated and stored at -20°C until further analysis. Lipid peroxidation was assessed by quantifying thiobarbituric acid reactive substances (TBARS). Aldehyde quantification was based on their extinction coefficient (ε532nm = 1.56 × 10^5 ^M^-1^ cm^-1^). Total non-enzymatic antioxidant capacity (TAC) was assessed spectrophotometrically using the ferric reducing antioxidant power assay (FRAP), following the methodology outlined [19]. Outcomes were Calculated using a standard curve generated from various concentrations of ferrous sulfate solutions. Superoxide dismutase (SOD) activity was measured spectrophotometrically at 420 nm by quantifying the inhibition of pyrogallol autoxidation in 50 mmol/L Tris-HCl buffer (pH 8.2) supplemented with 1 mmol/L diethylenetriamine penta-acetic acid. One unit of SOD was defined as the enzyme amount required to decrease pyrogallol autoxidation by 50%. All values were normalized to total protein content, determined by the Bradford method.

### Raman spectra analysis and data analysis: multidimensional projection

Raman spectra of blood serum from CT and T rats were obtained using a portable Raman spectrometer (Bruker, model BRAVO) with a Duo LaserTM system (785 and 852 nm) and SSETM (fluorescence mitigation). Raman spectra were obtained by adding 2 mL to a cuvette on the portable Raman holder. And area values at 417, 495, 646 and 1444 cm^-1^ were calculated using GRAMS/AI™ Spectroscopy Software [20].

### Fractal dimension

The fractal dimension of the tissue was evaluated in the urinary bladder stained with hematoxylin and Eosin (H&E) and picrosirius red. Images were captured at 400× magnification to assess nuclear and extracellular matrix disorganization. The ImageJ software (version 1.50i) was used, applying the fractal box-counting method as a two-dimensional approach, which quantifies the distribution of pixels in specific spatial areas. With this method, two images with the same pixel distribution (binarized and grayscale) have the same fractal dimension [21]. The calculated fractal dimension is always between 0 and 2, where values close to 2 represent greater tissue or cellular disorganization.

### Immunohistochemical analyzes

The slides underwent microwave treatment at 700–800 W, followed by incubation in sodium citrate buffer (0.01 M, pH 6.0). Endogenous peroxidase activity was inhibited by inhibited by exposure to a 3% hydrogen peroxide/methanol solution, while nonspecific binding was blocked by incubating the slides in a 3% Bovine Serum Albumin (BSA) solution. Subsequently, the sections were incubated overnight with primary antibodies against HIF1-α (1:100, sc-13515, Santa Cruz Biotechnology®), VEGF (1:100, C-1, sc-7269, Santa Cruz Biotechnology®), AT1 (1:100, G-3, sc-515884, Santa Cruz Biotechnology®), MAS1 (1:100, G-1, sc-390453, Santa Cruz Biotechnology®), NGF (1:100, sc-32300, Santa Cruz Biotechnology®), TGFβ1 (1:100, sc-130348, Santa Cruz Biotechnology®), SMAD2/3 (1:100, pa5-36125, Invitrogen®). Following this, sections were treated with anti-rabbit and/or anti-goat HRP secondary antibodies at a 1:200 dilution in 1% BSA. Diaminobenzidine (DAB) chromogen was used for visualization, and sections were counterstained with Harris hematoxylin. The intensity of HIF1-α, VEGF, AT1, MAS1, NGF, TGFβ1 and SMAD2/3 immunoreactivity was analyzed in 12 fields per animal using ImageJ software version 1.50i (National Institutes of Health, Bethesda, MD, USA). Immunostained areas were quantified in each image, and results were expressed as the percentage of the total analyzed area [22].

### Bioinformatics of public transcriptomic data

To confirm the role of fibrosis, angiogenesis, and hypoxia in bladder health, we analyzed publicly available datasets from bladders of animals with chronic ischemia and aging. The datasets used were obtained from the GeneNetwork database (www.genenetwork.org) and accessed on December 6, 2024. It is important to note that this study did not involve primary RNA extraction. All procedures related to RNA isolation, library preparation, and sequencing were performed by the original studies that generated these datasets, as described in their respective publications. Datasets were selected based on their relevance to bladder function under conditions of ischemia and aging, as well as the availability of RNA sequencing data. We performed a GO enrichment analysis of cellular components and biological processes in a rat model chronic bladder ischemia (GSE122060; *n* = 3 sham and 3 submitted to ischemia) [23]. Sixteen-week-old Sprague–Dawley rats were used, and bladder ischemia was induced by endothelial injury of the iliac arteries (AI-30, 30 injury cycles per iliac artery) the animals also received a 2% cholesterol diet to promote atherosclerosis. The sham group underwent a sham operation and received 2% cholesterol diet. After 8 weeks, the gene expression was analyzed. The ShinyGO software (ShinyGO 0.81) was used for bioinformatic analysis and graphical representation of the data based on edge cutoff = 0.2 and FDR *p*-value cutoff = 0.05, selecting we selected the *Rattus norvegicus* as the species.

Considering that the aging process has been shown to increase susceptibility to bladder dysfunction, we compared mRNA levels between old (Male Fischer 344 rats; 25–28 months-old) and young animals (6 months-old) fed *ad libitum* (GSE63650; *n* = 4 per group) [24]. Principal Component Analysis (PCA) was performed using with Metaboanalyst software (https://www.metaboanalyst.ca/). Cluster heatmaps and volcano plots were generated using the SRplot free online platform (https://www.bioinformatics.com.cn/en,). The fold enrichment of upregulated genes is presented in descending order based on enrichment results *p*  <  0.05. The *p*-value reflects the probability of observing specific proteins in an annotated protein list for a given term. Addicionally, we examined protein-protein interactions using bioinformatics tools via STRING Interactome 12.0 (Search Tool for the Retrieval of Interacting Genes/Proteins), avaliable at https://string-db.org using a medium confidence score (0.400).

### Statistical analysis

The Shapiro-Wilk test was used to assess the normality of all datasets. Data are presented as the mean ± standard deviation (SD). Statistical comparisons were made using Student’s T-test for unpaired samples. A paired t-test was performed to analyze the baseline (pre) and post-8 week (post) systolic blood pressure. All analyses were conducted using GraphPad Prism 10 software with a predetermined significance level of 5% (*p* < 0.05).

## Results

### HIIT training improves metabolic parameters

**Fig. 2:**
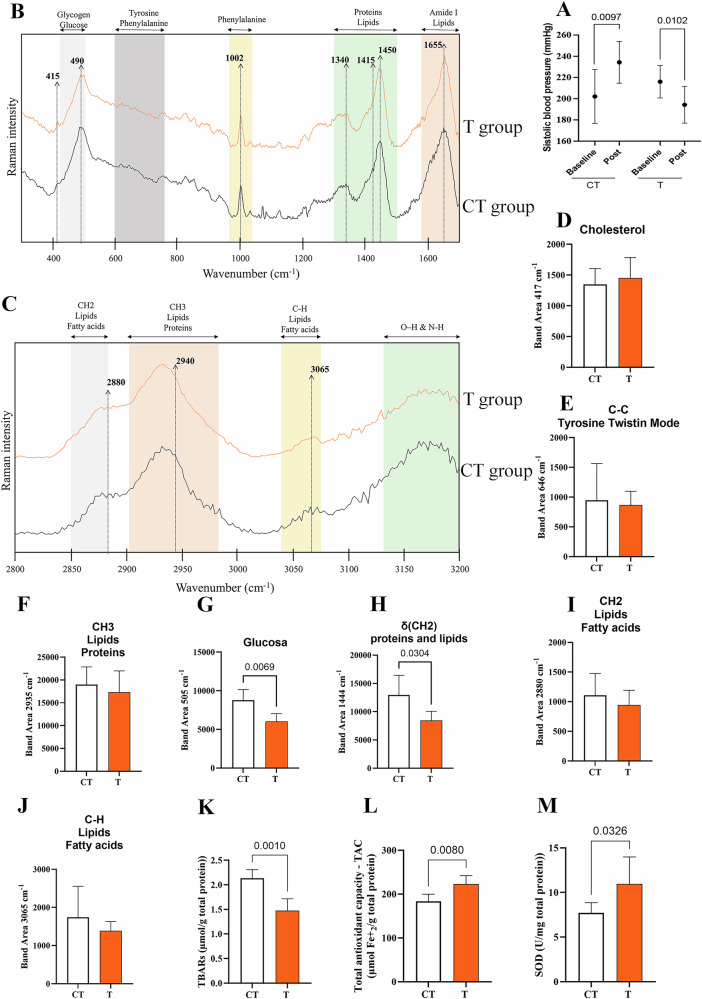
Systolic blood pressure, Raman spectral profile, and oxidative stress-related parameters in the serum of CT and T groups; **A** Systolic blood pressure measured before (Baseline) and after (Post) the experimental period in the control (CT) and trained (T) groups. **B** Representative Raman spectra in the fingerprint region (350–1700 cm⁻¹) obtained from serum samples of CT and T groups, highlighting the main vibrational bands assigned to glycogen/glucose, tyrosine/phenylalanine, phenylalanine, proteins/lipids, and amide I/lipids. **C** Representative Raman spectra in the high-wavenumber region (2800–3200 cm⁻¹), showing bands associated with CH₂ lipids/fatty acids, CH₃ lipids/proteins, C–H lipids/fatty acids, and O–H & N–H vibrations. Bar graphs represent the integrated band areas for **D** cholesterol, **E** C–C tyrosine twisting mode, **F** CH₃ lipids/proteins, **G** glucose/glycogen, **H** δ(CH₂) proteins and lipids, **I** CH₂ lipids/fatty acids, and **J** C–H lipids/fatty acids. Biochemical oxidative stress-related parameters are shown in **K** TBARS, **L** total antioxidant capacity (TAC), and **M** SOD activity. Data are expressed as mean ± SD. Statistical comparisons were performed using Student’s t-test, with *p* < 0.05 considered statistically significant

To assess the systemic pressor effect of HIIT, systolic blood pressure was evaluated. After 8 weeks, the control group exhibited a significant increase in systolic blood pressure compared to baseline (pre-intervention) (*p* = 0.0097, Fig. 2A). In contrast, the trained group subjected to HIIT showed an approximately Δ% -10 reduction in systolic blood pressure relative to baseline after 8 weeks of training (*p* = 0.0102; Fig. 2A). Analysis of the difference between Raman spectra was conducted using serum from CT rats and T rats. The Raman technique provides molecular-level information, enabling the investigation of functional groups, types of bonds, molecular conformations, and direct biochemical composition assessment (Fig. 2 B and C). Graphical evaluation of band areas revealed no significant differences between groups in the Cholesterol areas (Fig. 2D), as well as C-C twisting mode of tyrosine band area (Fig. 2E), CH2 of lipids and fatty acids band (Fig. 2I), CH3 of lipids and proteins (Fig. 2F), and C-H of lipids and fatty acids (Fig. 2J). The T group promotes significant reductions compared to the CT group in bands corresponding to Glucose 505 cm^-1^ (*p* = 0.0069; Fig. 2G) and δ(CH2) of proteins and lipids 1444 cm^-1^ (*p* = 0.0304, respectively, Fig. 2H).

Since lipid peroxidation contributes to ROS generation, we investigated whether chronic hypertension induces ROS production and assessed the effects of T group on this process. To do so, we evaluated TAC and FRAP levels in the plasma of both groups. The T group significantly increased TAC levels in the T group compared to the CT group (*p* = 0.0455, Fig. 2L). In cellular metabolism, antioxidant enzymes such as SOD play a crucial role in reducing oxidative stress. Consistent with the TAC findings, the T group exhibited significantly higher SOD activity than the CT group (*p* = 0.0257; Fig. 2M). Furthermore, our results showed a reduction in oxidative damage in the T group compared to the CT group, as measured by TBARS (*p* = 0.0278; Fig. 2K).

### HIIT training improves nuclear and collagen fractal dimension in urinary bladder

**Fig. 3:**
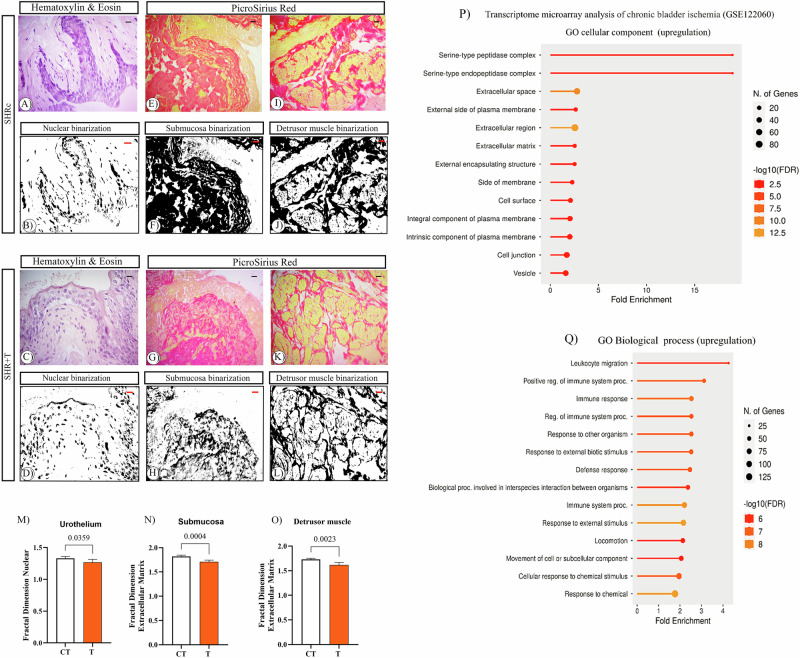
Histological and fractal dimension analysis of the H&E and PicroSirius Red techniques. **A–D** correspond to the H&E images and their respective fractal binarizations; **E–H** correspond to PicroSirius Red urothelial images and their respective fractal binarizations; **I–L** correspond to PicroSirius Red images of the detrusor muscle and their respective fractal binarizations. **M** Nuclear fractal density graphs; **N** fractal density graphs of the extracellular matrix of the urothelium and **O** fractal density graphs of the extracellular matrix of the detrusor muscle. GO cellular component **P** and molecular function **Q** enrichment of upregulated genes in chronic bladder ischemia model (GSE122060). The dot size corresponds to the number of genes under specific GO terms

Next, we evaluated the nuclear cellularity and collagen deposition in urinary bladder. As shown in Fig. 3, the T group exhibited a significantly lower mean fractal dimension of the urothelial nuclei compared to the CT group (*p* = 0.0369; Fig. 3B, D and M), indicating reduced nuclear cellularity in the urothelial layer.

Further analysis of the extracellular matrix components revealed that CT group displayed an increased fractal dimension, indicative of higher collagen deposition in both the submucosa and detrusor muscle (Figs. 3N and 3O). In contrast, animals subjected to T group demonstrated significantly reduced collagen deposition in both tissues (*p* = 0.0004 and *p* = 0.0023 for the submucosa and detrusor muscle, respectively), underscoring the effectiveness of HIIT in mitigating collagen accumulation.

### HIIT physical training improves urinary bladder angiogenic factors and reduces hypoxia

We observed an enrichment of serine-type peptidase and extracellular matrix (ECM) deposition in cellular components in transcriptome microarray analysis of chronic bladder ischemia (Fig. 3P). We also found an upregulation of genes related to immune and defense responses in the GO biological process analysis (Fig. 3Q). Animals with iliac arterial injury displayed 84 upregulated genes in the extracellular region (GO:0005576; FDR: 2.05e^-13^) and 2405 genes related to the immune system process (GO:0002376; FDR: 3.06e^-09^).

**Fig. 4:**
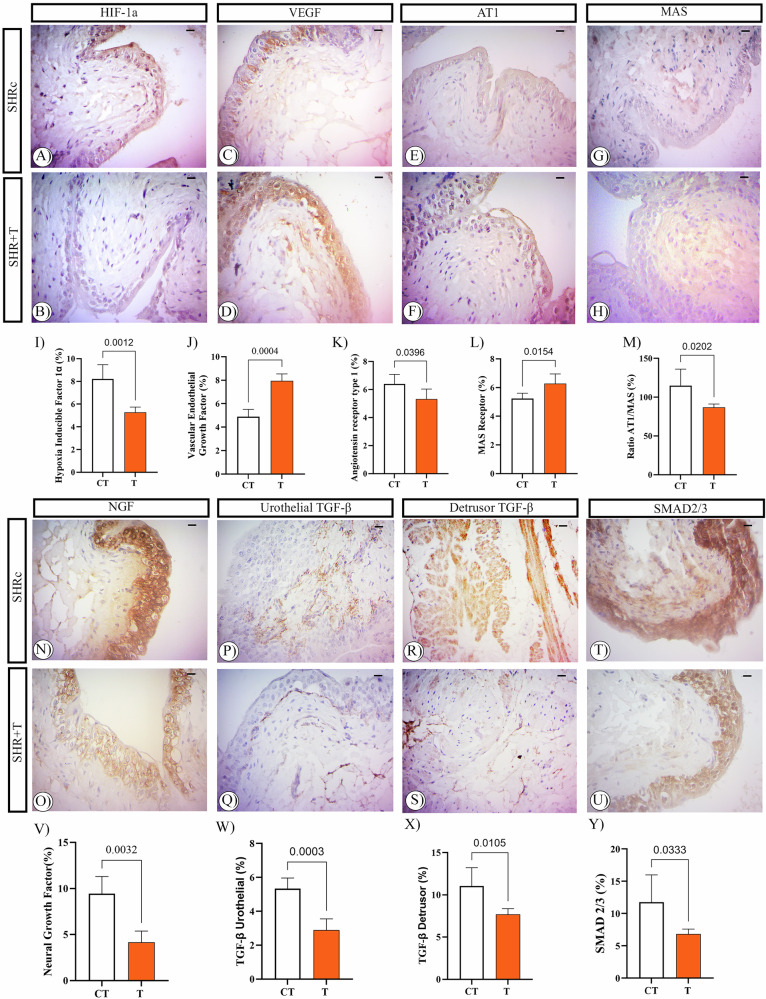
Immunohistochemistry analysis of the urinary bladder in hypertensive rats trained for 8 weeks. The upper images represent the SHRc group, while the lower images correspond to the SHR + T group. Images **A** and **B** show HIF-1α expression in the SHRc and SHR + T groups, respectively; **C, D** depict VEGF expression in the groups; **E, F** represent AT1 receptor expression in the CT and T groups, respectively; **G, H** show MAS receptor expression in the CT and T groups, respectively; **N, O** correspond to NGF immunostaining in the CT and T groups, respectively; **P, Q** illustrate TGF-β expression in the urothelial layer in the CT and T groups, respectively; **R, S** show TGF-β expression in the detrusor layer in the CT and T groups, respectively; and **T, U** represent SMAD2/3 immunostaining in the CT and T groups, respectively. The graphs provide quantitative analysis of the expressions: **I** graphical representation of HIF-1α expression; **J** VEGF expression; **K** AT1 receptor expression; **L** MAS receptor expression; **M** AT1/MAS receptor ratio; **V** NGF expression; **W** TGF-β expression in the urothelial layer; **Y** TGF-β expression in the detrusor layer; and **Z** SMAD2/3 expression. An unpaired t-test was used for group comparisons. Scale bar = 20 μm, 400x magnification. Data are expressed as mean ± SD

Adding to the knowledge about the vasoconstrictive effects of arterial hypertension in SHR animals, our study aimed to elucidate the protein expression levels of Hypoxia Inducible Factor 1 alpha (HIF-1α), a transcription factor responsive to low oxygen levels. Immunostaining analysis revealed greater HIF-1α protein expression in the urothelium of CT group compared to the T group (*p* = 0.0012; Fig. 4 A,B,I). Conversely, T group increased Vascular Endothelial Growth Factor (VEGF) protein expression compared to CT group (*p* = 0.0004; Fig. 4 C,D, J).

### HIIT control renin-angiotensin system receptors and neural activation of NGF

Inflammatory and angiogenic responses are tightly regulated by angiotensin II receptors, particulary the type 1 angiotensin receptor (AT1), which plays a crucial role in the dysregulation of urinary bladder and arterial contractility thereby exacerbating inflammation and collagen production [25, 26]. Our results reveal that the expression of AT1 receptors increased in the urinary bladder of the CT group compared to those subjected to T group (*p* = 0.0396; Fig. 4K), highlighting the effect of arterial hypertension on receptor modulation. In the other hand, the expression of the MAS receptor was increased in the T group compared to CT group (*p* = 0.0154; Fig. 4 G, H, M). Notably, T group reduced the AT1/MAS ratio compared to CT group (*p* = 0.0202; Fig. 4M), suggesting a possible regulatory effect of exercise on the balance of receptors in the renin-angiotensin system.

Given that angiotensin receptors play a crucial role in vascular and inflammatory regulation, their modulation by hypertension and exercise may also influence neural pathways involved in bladder dysfunction. In this context, we sought to investigate the impact of hypertension on neurotrophic factors in the urinary bladder. We found that T group significantly reduced urothelial NGF expression compared to CT group (*p* = 0.0032; Fig. 4N, O, and V).

### Arterial hypertension induces fibrotic remodeling of the detrusor, which is attenuated by HIIT through reduction of TGF-β expression

To assess the impact of arterial hypertension on detrusor remodeling, we examined the expression of key proteins involved in fibrosis. TGF-β1 levels were significantly reduced in the urothelial layer and detrusor muscle of T group (2.89 ± 0.66, *p* = 0.003, and 7.68 ± 0.68, *p* = 0.010, respectively) compared to CT group (5.33 ± 0.62 and 11.06 ± 2.16, respectively; Fig. 4W and Y). Similarly, its downstream target, SMAD2/3, showed decreased expression in the T group (6.82 ± 0.73) compared to the CT group (11.75 ± 4.22; *p* = 0.033, Fig. 4Z).

### Bioinformatics findings highlight TGF-β pathway, RAS activation, and hypoxia in bladder aging and fibrosis

**Fig. 5:**
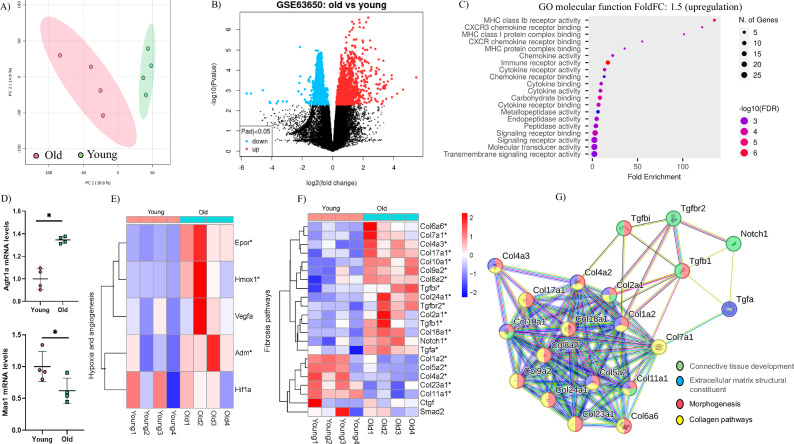
Bioinformatic of publicly available microarray dataset in the bladder during aging process (GSE63650). PCA analysis **A**, Volcano plot of gene expression regulated by aging **B**, GO molecular function enrichment of upregulated genes in the old group **C**, comparison of *AT1* and *MAS1* mRNA levels between different ages **D** Heat map of genes related to hypoxia, angiogenesis **E** and fibrosis pathways **F**, Protein-protein interaction analysis based on STRING network analysis with an interaction confidence score of 0.4 **G**. (*n* = 4 per group, * *p* ≤ 0.05 compared to young)

Results from the PCA are shown in Fig. 5A. This analysis aimed to identify gene expression patterns distinguishing the experimental groups. Notably, there is no overlap between the young and old groups. The variability in data captured by PCA along PC1 and PC2 was 36.6% and 14.8%, respectively. A total of 62,976 genes were identified by microarray analysis. The volcano plot (Fig. 5B) reveals 4,642 upregulated and 1,592 downregulated genes in the old group compared to young animals. Applying a threshold of 1.5-fold change and *p* = 0.05 in the t-test, GO molecular function enrichment analysis identified upregulated genes associated with cytokine activity (GO:0005125; FDR:0.0001) and metallopeptidase activity (GO:0008237; FDR: 0.0068) in the old group (Fig. 5C).

Interestingly, aged mice exhibited significantly higher mRNA levels of *Agtr1a* (*p* = 0.0004), while *Mas1* gene expression was reduced (*p* = 0.0489) (Fig. 5D), highlighting a relevant convergence between publicly available transcriptomic data and our immunohistochemical analyses. The aging process led to the upregulation of three genes associated with hypoxia pathways (*Epor*, *Hmox1*, and *Adm*), supporting a progressive increase in hypoxia-related dysfunctions. However, *Hif1a* and *Vegfa* expression levels remained unchanged (*p* > 0.05). Fibrosis-related pathways, aged animals exhibited increased mRNA expression of *Tgfa*, *Tgfb1*, *Tgfbi*, *Tgfr2*, and *Notch1*. The network of significantly upregulated genes related to connective tissue development, extracellular matrix structural constituent, morphogenesis, and collagen pathways consisting of 20 nodes and 112 edge an average node degree of 11.2. This high connectivity suggests a strong interplay between fibrosis and TGF-beta pathways.

## Discussion

In the present study, we investigated the effects of arterial hypertension on the urinary bladder microenvironment of spontaneously hypertensive rats (SHR) subjected to an 8-week HIIT protocol. Overall, our findings demonstrate that HIIT attenuated a systolic pressure and important molecular and structural alterations associated with hypertensive bladder remodeling. Specifically, trained animals exhibited improved circulating metabolic and redox parameters, reduced hypoxia-related signaling, increased VEGF expression, reduced urothelial NGF expression, attenuation of collagen deposition and TGF-β/SMAD2/3 signaling, as well as modulation of the local angiotensin receptor profile, characterized by reduced AT1 receptor expression, increased Mas receptor expression, and an improved AT1/Mas ratio. These results support the view that HIIT promoted a coordinated protective response against hypertension-induced bladder injury through integrated effects on oxidative balance, angiogenic signaling, fibrotic pathways, neural mediators, and local renin-angiotensin system regulation.

The 8-week duration of our intervention is consistent with evidence showing that clinically meaningful cardiovascular adaptations to HIIT can be achieved within this timeframe in hypertensive populations. HIIT is recognized as a time-efficient strategy capable of promoting rapid improvements in blood pressure, endothelial function, and cardiorespiratory fitness [27]. Results of meta-analytic data indicating that 6–12 weeks are sufficient to significantly reduce systolic and diastolic blood pressure [28]. Moreover, longer interventions further support the persistence of these adaptations, as demonstrated by sustained improvements in exercise-related blood pressure responses after 12 weeks of HIIT [29]. In agreement, our findings show that 8 weeks of HIIT significantly reduced systolic blood pressure compared to sedentary controls, with a greater delta reduction confirming the antihypertensive efficacy of the protocol. However, these benefits are dependent on continued training, as they may decline within weeks after detraining [30].

Our previous studies [18, 31], demonstrated that HIIT protocol induces cardiovascular and anthropometric adaptations in SHR, including reductions in heart rate, atrial and ventricular size modulations together with protection against inflammatory and structural damage in target organs such as the prostate and urinary bladder. These findings are consistent with previous evidence showing that HIIT exerts effects in angiotensin II-induced hypertensive models [32], and that training-induced cardiovascular adaptation may involve improved myocardial capillarization and hemodynamic regulation [33]. Within this framework, the present study substantially advances our previous observations by demonstrating that the protective effects of HIIT in the hypertensive bladder are not restricted to systemic cardiovascular improvement but also involve modulation of molecular pathways directly linked to oxidative balance, hypoxia, angiogenic signaling, neural mediators, profibrotic responses, and local angiotensin receptor regulation. Thus, the present findings strengthen the concept that HIIT acts not merely as a blood pressure-lowering intervention, but as a biologically relevant modulator of the bladder microenvironment under chronic hypertensive conditions.

Using Raman spectroscopy, we identified peaks at 417, 505, 646, 1444, 2880, 2935, and 3065 cm⁻¹, where the most altered Raman peaks were observed in both SHR and SHR + T. Using Raman analysis, we confirmed that led to a decrease in the area under the curve of the peak at 505 cm⁻¹, associated with glucose/glycogen vibrational bands, and at 1444 cm⁻¹, corresponding to the δ(CH₂) vibrational mode of lipids. Metabolic disorders are commonly associated with high blood pressure [34], a condition in which SHR animals have been reported to exhibit insulin resistance [34]. This phenotype disrupts the insulin signaling pathway, a key regulator of glucose and lipid metabolism, leading to significant difficulties in weight gain. The reduction in abdominal adiposity and serum lipids commonly observed as an adaptation to exercise training [35] is closely linked to improvements metabolic substrate utilization, including glucose and lipids. These adaptations enhance hepatic insulin sensitivity [36]. Additionally, exercise-induced changes in body composition, muscle physiology [37], and glucose metabolism [38], provide strong evidence of these beneficial effects. HIIT improves glucose uptake by skeletal muscle, increasing GLUT-4 content, and insulin sensitivity, which are triggered by muscle glycogen depletion [39]. Furthermore, in individuals with insulin resistance or diabetes, HIIT and resistance exercise promote the translocation of GLUT4, a muscle-specific glucose transporter, directly to the cell membrane [40]. This insulin-independent process allows for efficient glucose uptake, offering a significant advantage in glycemic control, especially in individuals with deficiencies in the insulin-dependent glucose transport mechanism, as observed in diabetes or in SHR animals that present insulin resistance [41]. The findings of the present study contribute to bridging the scientific gap regarding the beneficial effects of HIIT. We propose that the improvements in circulating biochemical parameters induced by HIIT make it a valuable non-pharmacological approach for the clinical management of arterial hypertension and metabolic diseases such as diabetes.

To explore the molecular events of oxidative stress associated with arterial hypertension, we analyzed the redox status in blood serum. Oxidative imbalance plays a crucial role in intracellular signaling pathways implicated in hypertension, including those mediated by MAPK and tyrosine kinases [42]. These pathways regulate essential cellular processes such as proliferation, migration, hypertrophy, inflammation, and fibrosis, all of which contribute to hypertensive pathophysiology [43]. In addition, the overproduction of reactive oxygen species (ROS) in arterial hypertension disrupts intracellular calcium homeostasis and mediates the effects of vasoactive agents such as angiotensin II, endothelin-1, and aldosterone [44]. The activation of these mediators induces ROS generation, which in turn activates transcription factors such as NF-κB, STAT, and AP-1, thereby promoting the expression of pro-inflammatory genes and cytokines [45]. In this context, SHR animals exhibit oxidative stress markers and heightened NF-κB expression, changes that have been associated with bladder dysfunction under hypertensive conditions [46]. Given the metabolic and cardiovascular benefits of HIIT discussed above, we next examined its impact on antioxidant defenses and oxidative damage in SHR. Our findings revealed greater oxidative damage in the hypertensive control group, as indicated by increased plasma TBARS levels, whereas HIIT significantly reduced lipid peroxidation while increasing SOD activity and total antioxidant capacity. Antioxidants such as SOD play a crucial role in neutralizing superoxide radicals, these results support the view that HIIT improves the systemic redox environment in hypertensive animals. Importantly, this effect is likely to be relevant to the urinary bladder, since oxidative stress may interact with inflammatory signaling, profibrotic mediators such as TGF-β, and angiotensin-dependent pathways, thereby contributing to tissue remodeling in hypertension.

Another critical consequence of arterial hypertension is tissue hypoxia in peripheral organs, including the prostate and urinary bladder. Hypoxia-inducible factor 1-alpha (HIF-1α) is the primary protein regulating cellular responses to hypoxia. Under normoxic conditions, HIF-1α is rapidly degraded, whereas in hypoxic conditions, it stabilizes and is upregulated [47]. In animal models of partial bladder outlet obstruction, hypoxia triggers the upregulation of genes such as Vegf, Flt-1, and Glut-1, leading to disrupted glucose metabolism in the detrusor muscle, ultimately causing degeneration and necrosis of smooth muscle tissue [48]. In the urinary bladder, elevated HIF-1α expression induces pathological changes, including fibrosis and increased bladder wall stiffness. HIF-1α knockdown in bladder epithelial cell cultures not only alleviated hypoxia-induced cell death but also attenuated fibrosis [49]. These findings are in line with the present study, which demonstrates that arterial hypertension decreases VEGF expression and compromises bladder vascularization, which we can hypothesize as a possible reduction in blood supply and induction of hypoxia. This hypoxic state contributes to damage in the detrusor muscle and the bladder urothelium [50]. On the other hand, physical exercise has been reported to modulate angiogenesis through VEGF, improving muscle and peripheral capillarization, particularly in aged mice [51]. Consistently, our study shows that HIIT increased VEGF expression in the urothelium while reducing HIF-1α expression in SHR. These findings support the potential role of exercise in promoting tissue-specific angiogenic adaptations in the urinary bladder, improving blood supply, and mitigating the progression of hypoxia-induced tissue damage associated with hypertension.

Arterial hypertension is commonly associated with detrusor muscle hyperactivity. SHR animals exhibit increased levels of inflammatory bladder mediators, such as IL-6 and TNF-α, which contribute to bladder hyperactivity through reduced blood flow and increased oxidative stress in bladder tissue [43]. In parallel, hypertension-related bladder dysfunction has also been associated with increased levels of nerve growth factor (NGF), a key mediator of pathology-induced changes in C-fiber afferent excitability and bladder reflex activity [52]. In this context, the reduction in urothelial NGF expression observed after HIIT suggests that exercise may attenuate neural signaling pathways involved in hypertensive bladder dysfunction. This interpretation is consistent with the broader protective profile identified in the present study, in which HIIT was associated not only with lower NGF expression, but also with improvements in oxidative balance, hypoxia-related signaling, and fibrotic remodeling. Thus, the modulation of NGF by exercise may represent one component of a coordinated protective response against bladder injury induced by chronic arterial hypertension.

In arterial hypertension, particularly in the SHR model, the renin–angiotensin system becomes dysregulated, favoring activation of the classical ACE/Ang II/AT1R axis and contributing to vasoconstriction, oxidative stress, inflammation, fibrosis, and tissue remodeling [53] Angiotensin II is a potent vasoconstrictor [53]. In this context, angiotensin II is a potent vasoconstrictor, and AT1 receptor activation is associated with MAPK/ERK1-2/p38 signaling, vascular remodeling, and profibrotic responses in the urinary bladder. Importantly, experimental evidence indicates that AT1 receptors predominantly mediate Ang II-induced contractile responses in rat bladder smooth muscle [53]. In addition, angiotensin II has been described as a trophic factor in the bladder wall, promoting smooth muscle hypertrophy/hyperplasia and increased collagen production, which further strengthens the biological link between AT1 activation and bladder remodeling under chronic hypertensive conditions [54]. In contrast, the Mas receptor, through the ACE2/Ang-(1-7)/MasR pathway, exerts counter-regulatory actions that are generally associated with vasodilation, anti-inflammatory signaling, and antifibrotic effects [55] Thus, the increase in AT1 receptor expression together with the reduction in Mas receptor expression observed in hypertensive animals can be interpreted as a maladaptive shift of the local bladder angiotensin system toward its deleterious arm. Within this framework, the present findings are particularly relevant because HIIT reduced AT1 receptor expression, increased Mas receptor expression, and improved the AT1/Mas ratio, suggesting that exercise was able to counteract the angiotensin receptor imbalance induced by hypertension and restore a profile more compatible with tissue homeostasis. This interpretation is consistent with previous evidence showing that exercise training can modulate RAS signaling under hypertensive and cardiometabolic conditions, favoring restoration of the balance between the classical and counter-regulatory axes [55–57]. Therefore, rather than representing an isolated receptor change, the modulation of AT1R/MasR observed here likely reflects an important mechanism through which HIIT attenuates bladder remodeling in arterial hypertension.

Arterial hypertension is a chronic condition that promotes progressive fibrotic remodeling, characterized by excessive extracellular matrix (ECM) accumulation in affected tissues. In the urinary bladder, previous studies have shown that SHR animals exhibit increased collagen deposition and marked ECM remodeling, supporting the concept that fibrosis is a relevant component of hypertensive bladder injury. Within this context, TGF-β1 is particularly important because it is widely recognized as a central regulator of fibrotic responses in cardiovascular and renal disease [58]. After activation, TGF-β1 phosphorylates Smad proteins, which translocate profibrotic signals to the nucleus and induce the expression of genes involved in matrix accumulation and tissue remodeling [59]. Phosphorylated Smad2/3 complexes play a decisive role in driving fibrotic progression [60] and dysregulation of this pathway has also been associated with interstitial fibrosis in neurogenic bladder conditions [61]. In addition, in vitro studies in human bladder smooth muscle cells have shown that TGF-β1 not only increases the expression of collagen types I and III, but also induces smooth muscle cell hypertrophy, reinforcing the functional relevance of this pathway to bladder remodeling [62]. Importantly, this profibrotic axis does not appear to act in isolation. Experimental evidence from other fibrotic models indicates that Ang II/AT1 signaling can stimulate TGF-β1 expression and extracellular matrix production, supporting a mechanistic interaction between the local angiotensin system and TGF-β-dependent remodeling [63, 64]. In the context of the present study, hypertensive animals exhibited increased TGF-β1 and SMAD2/3 expression together with greater collagen deposition, whereas HIIT attenuated all of these alterations. Thus, our findings support the view that exercise blunted a profibrotic signaling program in the hypertensive bladder, likely limiting ECM accumulation and structural remodeling through coordinated effects on pathways related to fibrosis, oxidative stress, and local angiotensin receptor imbalance.

Using bioinformatics tools, we identified a significant upregulation of genes associated with ECM organization, cell surface, and plasma membrane in chronic bladder ischemia, reinforcing the concept that ECM deposition is a hallmark of bladder dysfunction [65] Notably, aged animals exhibited increased mRNA expression of Agtr1a, along with a reduction in Mas1 gene expression, highlighting a dysregulated balance in the angiotensin signaling pathway that may contribute to vascular dysfunction and fibrosis as previoulsy described [66]. In parallel, the aging process led to the upregulation of hypoxia-related genes, including *Epor, Hmox*1, and *Adm*, suggesting a progressive deterioration of tissue oxygenation. However, no significant changes were observed in *Hif1a* and *Vegfa* mRNA expression, indicating that other molecular mechanisms may be driving the hypoxic response in aged bladders. [66]. Regarding fibrosis-related pathways, we found increased expression of key pro-fibrotic genes such as *Tgfa, Tgfb1, Tgfbi, Tgfr2*, and *Notch* genes further supporting the involvement of TGF-β signaling in the development of bladder fibrosis. Additionally, an enrichment of MMPs was detected in the urinary bladder of aged animals, a finding commonly associated with afferent nerve hyperexcitability, urinary dysfunction, and progressive bladder diseases. Given the role of MMPs in tissue remodeling, hypertrophy, angiogenesis, inflammation, and collagen turnover, their increased expression aligns with the fibrotic profile observed in aged and hypertensive conditions [67]. Importantly, the network analysis revealed a highly interconnected gene cluster related to connective tissue development, extracellular matrix structural components, morphogenesis, and collagen pathways, underscoring the intricate molecular interactions driving bladder fibrosis. The overlap between transcriptomic data and our histological findings strengthens the hypothesis that TGF-β signaling plays a central role in fibrotic remodeling of the aging bladder [66].

While our findings provide relevant mechanistic insight into hypertensive bladder remodeling, some limitations should be considered when interpreting the present data. First, although structural and molecular changes were consistently demonstrated, functional assessments such as voiding behavior, intravesical pressure, cystometry, or ex vivo contractility were not included and would be important to further define the physiological relevance of these alterations. The present analyses were performed in whole bladder tissue, which precludes cell-specific interpretation of the molecular changes observed and does not allow distinction among contributions from the urothelium, detrusor smooth muscle, fibroblast/interstitial populations, or vascular compartments. In addition, we focused on the local bladder angiotensin axis and did not perform a full systemic RAAS characterization, including circulating or tissue levels of Ang II, Ang-(1-7), ACE, and ACE2, which would have strengthened the mechanistic interpretation of the receptor imbalance identified here. Furthermore, although the public transcriptomic datasets were useful as complementary exploratory support, they were not derived from the animals used in the present study and should not be interpreted as substitutes for matched in vivo controls.

This study demonstrates that HIIT exerts a broad protective effect against hypertension-induced urinary bladder remodeling in SHR. In addition to improving metabolic and redox parameters, HIIT modulated key molecular pathways related to hypoxia, fibrosis, neural activity, and local angiotensin signaling. The reduction in HIF-1α, NGF, TGF-β1, and SMAD2/3 expression, together with decreased collagen deposition, increased VEGF expression, and improvement of the AT1/Mas receptor balance, indicates that exercise attenuated a maladaptive bladder phenotype induced by chronic arterial hypertension. Taken together, these findings expand the biological relevance of HIIT beyond systemic cardiovascular control and highlight its potential to modulate tissue-specific mechanisms involved in hypertensive bladder injury. Future studies integrating functional, cell-specific, and long-term approaches will be important to further define the physiological and translational implications of these adaptations.

## Supplementary information

Supplementary information

## Data Availability

The datasets used and/or analyzed during the present study come from procedures with animals and can be requested without restrictions from the corresponding author.
