# Supplementary material for: High-intensity interval training attenuates urothelial nerve growth factor and angiotensin axis in hypertensive urinary bladder
Source: Hypertens Res. 2026 May 20;49(7):2215–31. doi: 10.1038/s41440-026-02680-y (PMC13333501; doi:10.1038/s41440-026-02680-y)
Supplement: Supplementary file 1 — Supplementary information [file 41440_2026_2680_MOESM1_ESM.docx]

**1. SUPPLEMENTARY METHODS**

**1.1 Supplementary analysis of immunohistochemical protein-expression ratios**

To further investigate the interplay among inflammatory, angiogenic, hypoxic, and renin–angiotensin system-related pathways in the urinary bladder of spontaneously hypertensive rats (SHR), additional analyses were performed using ratios derived from immunohistochemical expression data. Immunohistochemical procedures and image acquisition were conducted as previously described by Veras et al. (2020). Semi-quantitative protein expression was obtained through image analysis based on the relative area of positive staining, and values were used to compute protein-to-protein ratios. This approach allows an integrative assessment of pathway balance, providing insight into the relative predominance of pro- versus anti-inflammatory, pro- versus anti-fibrotic, and vasoconstrictive versus vasodilatory signaling within the tissue microenvironment.

For each animal, ratios were calculated using the following formula:

Ratio (%) = (protein A / protein B) × 100

For example, the IL-6/IL-10 ratio was determined by dividing the immunohistochemical value of IL-6 by that of IL-10 and multiplying by 100. The same approach was applied to the following ratios: IL-6/IL-10, NF-κB/IL-10, MAS/IL-6, MAS/IL-10, MAS/TGF-β, MAS/VEGF, MAS/HIF-1α, AT1/IL-10, AT1/IL-6, AT1/TGF-β, AT1/VEGF, and AT1/HIF-1α.

Data are presented as mean ± standard deviation (SD). Statistical comparisons between the control hypertensive group (CT) and the trained hypertensive group (T) were performed using Student’s t-test, with significance set at p < 0.05.

**2. SUPPLEMENTARY RESULTS**

**2.1 Exercise training modifies immunohistochemical protein-expression ratios in the urinary bladder of spontaneously hypertensive rats**

To further evaluate the impact of exercise training on the balance among inflammatory, angiogenic, hypoxic, and renin–angiotensin system-related markers, protein-to-protein immunohistochemical expression ratios were analyzed in the urinary bladder of spontaneously hypertensive rats. The IL-6/IL-10 ratio was significantly reduced in the trained group (T) (65.60 ± 12.25) compared to the control group (CT) (105.9 ± 21.07; p = 0.0089; Supplementary Figure 1A). Similarly, the NF-κB/IL-10 ratio was lower in the T group (52.53 ± 14.12) than in the CT group (82.22 ± 4.89; p = 0.0069; Supplementary Figure 1B).

In contrast, ratios involving the Mas receptor indicated a shift toward a protective profile. The MAS/IL-6 ratio was significantly higher in the T group (116.2 ± 14.01) compared to the CT group (76.03 ± 28.40; p = 0.0307; Supplementary Figure 1C), while no difference was observed for MAS/IL-10 (p = 0.9421; Supplementary Figure 1D). The MAS/TGF-β ratio was markedly increased in trained animals (222.7 ± 63.19 vs. 106.5 ± 10.28; p = 0.0138; Supplementary Figure 1E), whereas the MAS/VEGF ratio was significantly reduced (72.42 ± 16.05 vs. 118.2 ± 28.84; p = 0.0200; Supplementary Figure 1F). Additionally, the MAS/HIF-1α ratio was significantly elevated in the T group (116.6 ± 15.83 vs. 70.10 ± 13.69; p = 0.0012; Supplementary Figure 1G).

Regarding AT1-related ratios, the AT1/IL-10 ratio was significantly reduced in the T group (65.36 ± 13.95) compared to the CT group (85.97±10.67; p=0.0323; Supplementary Figure 1H), while no significant differences were observed for AT1/IL-6 (p = 0.2852; Supplementary Figure 1I) or AT1/TGF-β (p = 0.0629; Supplementary Figure 1J). In contrast, the AT1/VEGF ratio was significantly lower in trained animals (62.86 ± 14.21 vs. 131.5 ± 12.18; p<0.0001; Supplementary Figure 1K), whereas the AT1/HIF-1α ratio was significantly higher in the T group (100.9 ± 11.99 vs. 78.58 ± 7.53; p = 0.0103; Supplementary Figure 1L).

These findings indicate that HIIT promotes a coordinated shift toward an anti-inflammatory and protective molecular profile in the hypertensive bladder, characterized by attenuation of pro-inflammatory signaling, modulation of angiogenic and hypoxic pathways, and rebalancing of the renin-angiotensin system toward a more favorable axis.


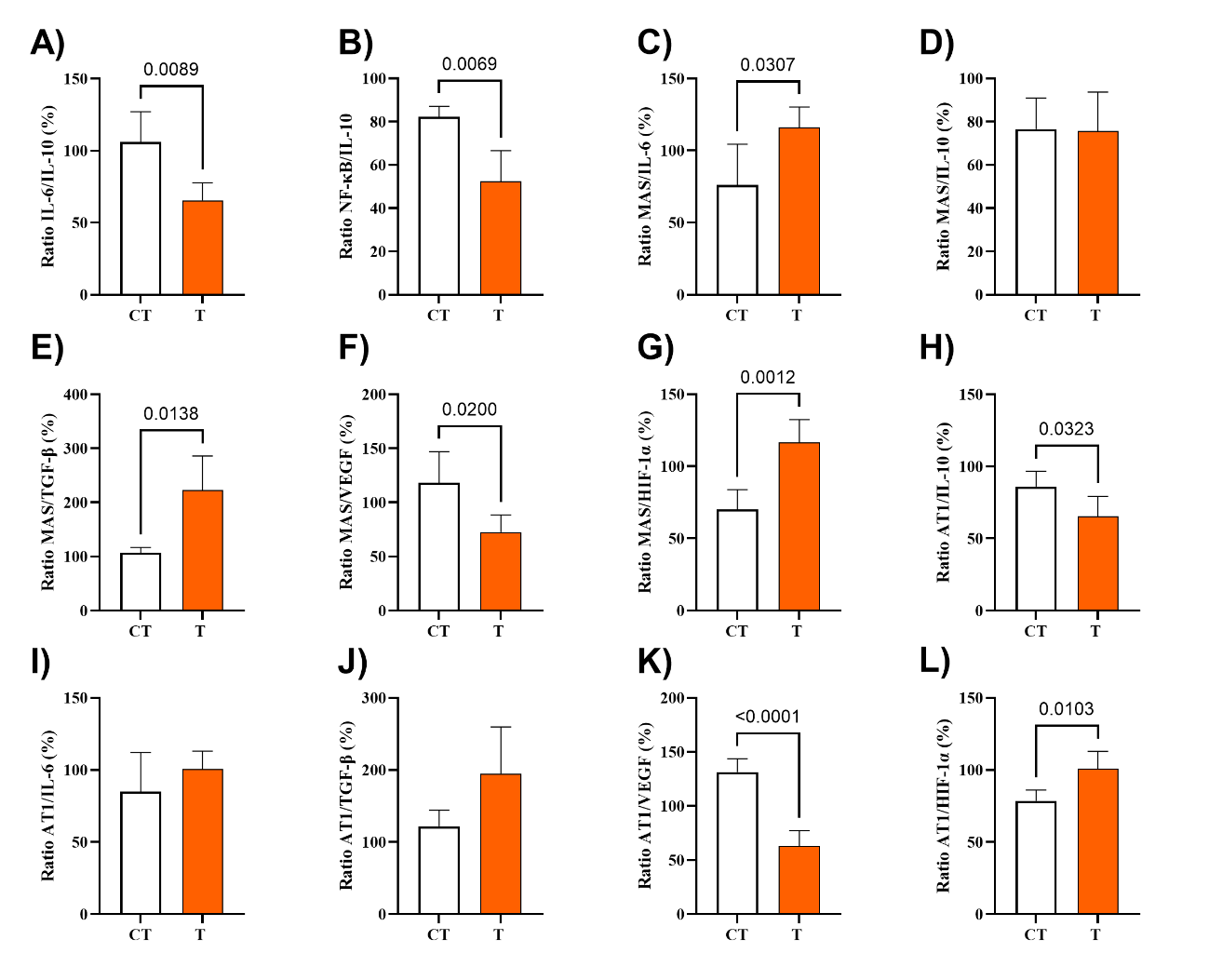


**Supplementary Figure 1. Protein-to-protein immunohistochemical expression ratios in the urinary bladder of spontaneously hypertensive rats (SHR):** Bar graphs show the relative ratios between inflammatory, angiogenic, hypoxic, and renin–angiotensin system-related proteins in the urinary bladder of the control hypertensive group (CT) and the trained hypertensive group (T). Ratios were calculated according to the equation (protein A / protein B) × 100. Panels represent the following ratios: (A) IL-6/IL-10, (B) NF-κB/IL-10, (C) MAS/IL-6, (D) MAS/IL-10, (E) MAS/TGF-β, (F) MAS/VEGF, (G) MAS/HIF-1α, (H) AT1/IL-10, (I) AT1/IL-6, (J) AT1/TGF-β, (K) AT1/VEGF, and (L) AT1/HIF-1α. Data are expressed as mean±SD. Statistical comparisons between groups were performed using Student’s t-test, with significant differences indicated by the corresponding p values shown above the bars.

References

Veras, A.S.C., de Freitas, M.C., Thorpe, H.H.A. et al. Strength Training Modulates Prostate of Wistar Rats Submitted to High-Fat Diet. Reprod. Sci. 27, 2187–2196 (2020). https://doi.org/10.1007/s43032-020-00238-y
